# Genomic characterisation of the effector complement of the potato cyst nematode *Globodera pallida*

**DOI:** 10.1186/1471-2164-15-923

**Published:** 2014-10-23

**Authors:** Peter Thorpe, Sophie Mantelin, Peter JA Cock, Vivian C Blok, Mirela C Coke, Sebastian Eves-van den Akker, Elena Guzeeva, Catherine J Lilley, Geert Smant, Adam J Reid, Kathryn M Wright, Peter E Urwin, John T Jones

**Affiliations:** The James Hutton Institute, Dundee Effector Consortium, Invergowrie, Dundee, DD2 5DA UK; Centre for Plant Sciences, University of Leeds, Leeds, LS2 9JT UK; A.N. Severtsov Institute of Ecology and Evolution, Russian Academy of Sciences, Leninskii Prospect 33, Moscow, 119071 Russia; Laboratory of Nematology, Department of Plant Sciences, Wageningen University, Droevendaalsesteeg 1, 6708 PB Wageningen, The Netherlands; Wellcome Trust Sanger Institute, Wellcome Trust Genome Campus, Cambridge, CB10 1SA UK

**Keywords:** Plant parasitic nematode, Effector, Genome, Transcriptome

## Abstract

**Background:**

The potato cyst nematode *Globodera pallida* has biotrophic interactions with its host. The nematode induces a feeding structure – the syncytium – which it keeps alive for the duration of the life cycle and on which it depends for all nutrients required to develop to the adult stage. Interactions of *G. pallida* with the host are mediated by effectors, which are produced in two sets of gland cells. These effectors suppress host defences, facilitate migration and induce the formation of the syncytium.

**Results:**

The recent completion of the *G. pallida* genome sequence has allowed us to identify the effector complement from this species. We identify 128 orthologues of effectors from other nematodes as well as 117 novel effector candidates. We have used *in situ* hybridisation to confirm gland cell expression of a subset of these effectors, demonstrating the validity of our effector identification approach. We have examined the expression profiles of all effector candidates using RNAseq; this analysis shows that the majority of effectors fall into one of three clusters of sequences showing conserved expression characteristics (invasive stage nematode only, parasitic stage only or invasive stage and adult male only). We demonstrate that further diversity in the effector pool is generated by alternative splicing. In addition, we show that effectors target a diverse range of structures in plant cells, including the peroxisome. This is the first identification of effectors from any plant pathogen that target this structure.

**Conclusion:**

This is the first genome scale search for effectors, combined to a life-cycle expression analysis, for any plant-parasitic nematode. We show that, like other phylogenetically unrelated plant pathogens, plant parasitic nematodes deploy hundreds of effectors in order to parasitise plants, with different effectors required for different phases of the infection process.

**Electronic supplementary material:**

The online version of this article (doi:10.1186/1471-2164-15-923) contains supplementary material, which is available to authorized users.

## Background

Plant-parasitic nematodes (PPN) cause severe damage to crops throughout the world and are an important constraint on delivering food security. Establishing the value of damage and control costs for these pathogens is difficult but has been calculated as being in excess of US$80 billion each year [1]. The largest economic losses are caused by the sedentary endoparasitic root-knot and cyst nematodes of the genera *Meloidogyne* and *Heterodera/Globodera* [2]. These nematodes have complex, biotrophic interactions with their hosts and induce the formation of feeding structures from which they derive all the food required for development. The feeding site needs to be maintained and protected from host defence responses, as well as from potential pathogens, for several weeks while the nematode matures to the adult stage.

The white potato cyst nematode, *Globodera pallida*, is an important pathogen of potato wherever it is grown. *Globodera pallida* originated in South America [3] and was introduced into Europe in the 19^th^ century with wild potato material used for resistance breeding against late blight. It is now widely distributed in Europe and in regions that have imported seed potato from Europe [4]. Yield losses in excess of 50% have been reported and the lack of major gene resistance for *G. pallida,* coupled to the increasing legislative restrictions on the use of nematicides, mean that new control strategies are required.

*Globodera pallida* hatches as a second stage juvenile (J2) in response to diffusates from roots of suitable host plants. The J2 then locates the root, invades and migrates destructively through root cells until it reaches the inner cortex layers. During this migration the nematode uses the stylet to mechanically disrupt host cells and releases a cocktail of plant cell wall degrading enzymes that soften the cell wall [5]. At the inner cortex the behaviour of the nematode changes and it probes individual cells with the stylet until a cell that does not respond adversely is detected (reviewed in [6]). The nematode then secretes proteins into this initial syncytial cell which is transformed into a large multinucleate syncytium. Cell wall openings are formed, initially by widening of pre-existing plasmodesmata, followed by controlled breakdown of the plant cell wall in these regions. The cytoplasm of the initial syncytial cell proliferates, the central vacuole breaks down and the nucleus becomes enlarged. These changes are also observed in the cells surrounding the initial syncytial cell which implies communication between these cells. Eventually the protoplasts of the initial syncytial cell and its neighbours fuse at the cell wall openings and this process is repeated with further layers of cells until 200–300 cells are incorporated into the syncytium. Each nematode can only create one syncytium and depends on it for all the nutrients required for development to the adult stage, a process that takes 4–6 weeks. This prolonged period of biotrophy is almost unparalleled in plant-pathogen interactions and demonstrates a clear need for the nematode to suppress host defences throughout the life cycle.

The interactions of PPN, including *G. pallida*, are mediated by effectors – defined broadly here as secreted nematode proteins that manipulate the host to the benefit of the nematode. Effectors of PPNs are mainly secreted from two sets of gland cells, the dorsal and subventral gland cells, through the stylet into the host. These sets of gland cells show distinct developmental profiles. The two subventral gland cells are large and full of secretory granules in invasive stage J2, decrease in size and activity during the sedentary parasitic stages but become active again in adult male nematodes, which leave the root in order to locate females. In contrast, the dorsal gland cell is small in J2 but increases in size and activity in the sedentary stages [7]. It has therefore been widely suggested that proteins produced in the subventral gland cells are important for invasion, migration and processes occurring at the early stages of parasitism whereas those produced in the dorsal gland cell play a role in the later stages of the parasitic process.

Because of their key roles in parasitism, effectors of plant parasitic nematodes have been studied in detail. Effectors from cyst nematodes have been identified through EST sequencing *e.g.* [8], expression profiling [9] and, in particular, through sequencing of mRNA extracted from aspirated gland cell cytoplasm [10]. More recently, a protocol for purification of oesophageal gland cells and sequencing of purified mRNA has been described [11]. In each case, confirmation that a sequence of interest represents a candidate effector is obtained by *in situ* hybridisation to demonstrate expression in the subventral or dorsal oesophageal gland cells [12]. Following on from these projects, many effectors have been the subject of further functional characterisation. In addition to the cell wall modifying enzymes described above, effectors that play a wide variety of roles at various stages in the host parasite interaction have been described (reviewed in [13]. Effectors that suppress PAMP triggered immunity (PTI) and Effector triggered immunity (ETI) [14] have recently been identified from both cyst nematodes and root-knot nematodes [15, 16]. Other effectors may have roles in syncytium induction, either by targeting host auxin transporters [17] or by mimicking plant CLAVATA3 peptides [18]. An effector has also been identified that may play a role in the extensive remodelling of the host cell walls that takes place in the syncytium through its interaction with a host pectin methylesterase [19]. In other cases the host target of an effector has been identified along with a demonstration that the presence of the effector is of benefit to the nematode, although the precise function of the effector may remain uncertain (*e.g.* [20]).

Although enormous progress has clearly been made in terms of unravelling the functions of some nematode effectors, little genome scale analysis of the effectors present in any PPN has yet been reported. The genomes of two root knot nematodes, *Meloidogyne incognita* and *M. hapla* have been sequenced and some of the effectors present in each of these genomes were analysed as part of these projects [21, 22]. Further publications have described the large scale analysis of secreted proteins collected from one of these species [23, 24]. In addition, the genome and the secretome of a migratory endoparasitic nematode, *Bursaphelenchus xylophilus* have been sequenced [25, 26]. In contrast to cyst nematodes, *B. xylophilus* is not a biotrophic pathogen (in that it does not need to keep its host alive during the feeding process) and the effectors identified in this species were substantially different from those present in other PPN, with the exception of pectate lyases and expansins. This is likely to be a reflection of both the different modes of parasitism between *B. xylophilus* and cyst/root-knot nematodes and the taxonomic distance between them.

We recently reported the full genome sequence of *G. pallida* [27] including a fully replicated life cycle transcriptome analysis and a preliminary description of the effectors likely to be present in this species. Here we report the detailed genome scale analysis of the effector complement present in *G. pallida*, including analysis of expression profiles across the life cycle. We also examine the location of various effector gene families within the genome. In addition, we demonstrate the gland cell expression of effector candidates and in functional studies show that the effectors of *G. pallida* target a wide range of host subcellular compartments.

## Results & discussion

### Cell wall modifying proteins

**Figure 1:**
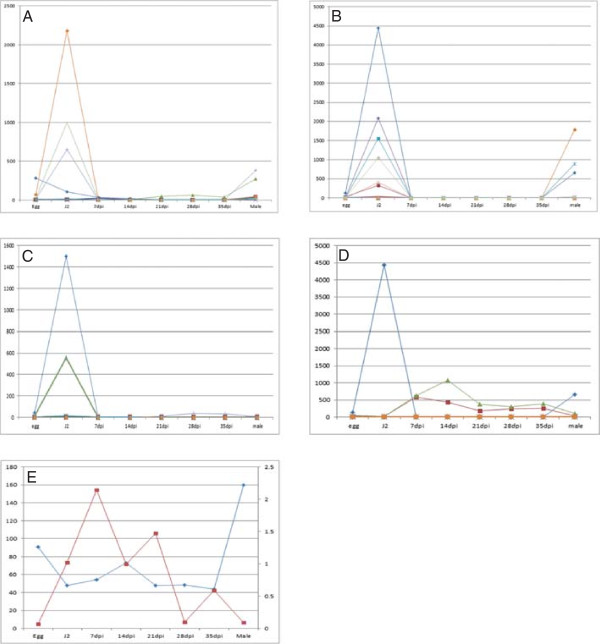
**Expression profiles of cell wall modifying proteins across the** ***Globodera pallida*** **life cycle.** Life stages indicated on X axis, Y-axis indicates reads per kilobase per million. Each line represents the expression profile for an individual gene. **(A)** Expansins: Expression profiles of nine sequences are shown. GPLIN_000293700 (orange line), GPLIN_000536200 (darker green line) and GPLIN_000599100 (light blue line) are upregulated at J2 and adult male stages while the other six sequences (GPLIN_000092400, GPLIN_000293400, GPLIN_000590900, GPLIN_000599200, GPLIN_001571600 and GPLIN_001621500) are only weakly expressed at the life stages tested; **(B)** Cellulases: Expression profiles of fifteen sequences are shown. One sequence GPLIN_000536400 – dark blue) is upregulated at J2 and male, five sequences (GPLIN_001111200 – purple, GPLIN_001111300 – light blue, GPLIN_001215600 – light olive, GPLIN_000313600 – pink and GPLIN_000552400 – brick red) are upregulated at J2, two sequences (GPLIN_000755200 – orange, and GPLIN_000755100 – light blue) are upregulated at male only. The remaining cellulase-like sequences (GPLIN_000616300, GPLIN_000694900, GPLIN_000779000, GPLIN_779200, GPLIN_000827200, GPLIN_001185800 and GPLIN_000304900) are weakly expressed at the life stages shown here; **(C)** Pectate Lyases: Expression profiles of seven sequences are shown. Three sequences (GPLIN_000467400 – blue line, GPLIN_000142600 and GPLIN_000412300 – overlaying graphs appearing green in the figure) are upregulated at J2 while four others (GPLIN_000673000, GPLIN_000294400, GPLIN_000294500 and GPLIN_000322300) are weakly expressed at the stages shown here; **(D)** Carbohydrate Binding Modules; **(E)** GH53 arabinogalactan endo 1,4 ß galactosidases. For Figure 1 **E** the left hand Y axis shows scale of expression for GPLIN_000142900 (blue line) while the right hand Y axis shows scale of expression for GPLIN_000143000 (red line).

PPN are almost unique among animals in having the capacity to metabolise the plant cell wall using endogenous cell wall degrading and modifying enzymes. The first effectors identified from PPN were cellulases, secreted from the subventral gland cells of cyst nematodes [28]. The similarity of these genes to bacterial cellulases and their absence from other animals led to the suggestion that they had been acquired from bacteria by horizontal gene transfer. Subsequent studies have led to the identification of numerous other horizontally acquired cell wall modifying proteins in a wide range of PPN, including cellulases, pectate lyases, xylanases, polygalacturonases, arabinogalactan galactosidases, arabinanases, carbohydrate binding modules (CBM) and expansin-like proteins (reviewed in [5]). The genome project for *Meloidogyne incognita* showed that more than 90 genes from seven protein families are present that could be involved in metabolism of the cell wall ([21] – Table 1). The *M. hapla* genome contains fewer genes albeit encoding a similar range of proteins [22], while non-plant parasites such as *Caenorhabditis elegans* or *Brugia malayi* have none (Table 1). The only noted exception to this rule is the presence of sequences similar to GH5 cellulases in *Pristionchus pacificus* [29] and these are thought to be part of a subclass of these enzymes that is distinct from those of the plant parasitic species. The *G. pallida* genome also contains a large number of genes that could encode cell wall modifying proteins ([27]; Table 1). The availability of the *G. pallida* transcriptome sequences allowed us to examine the expression profiles of such genes across the life cycle. The J2 (which needs to invade the host) and adult male (which needs to leave the root to locate females at the root surface) are the stages of the nematode that migrate through host tissues and many of the genes encoding cell wall modifying proteins, including a subset of the expansins and cellulases, were expressed specifically at these two life stages (Figure 1A and B). The pectate lyases that had detectable expression in the life stages tested were largely restricted to the J2 stage (Figure 1C), possibly also reflecting a role in migration. Some of the cellulases and expansins of *G. pallida* consist of a catalytic domain fused to a CBM. Like other PPN, *G. pallida* also has genes encoding proteins consisting of the CBM domain alone. All CBM protein domains present in *G. pallida* and other PPN, regardless of whether they are “stand alone” proteins or fused to cellulases or expansins are from CBM family 2. Some of these CBM proteins (not fused to any catylytic domain) are thought to be involved in migration. However, in *H. glycines* one CBM protein has been shown to interact with a host pectin methylesterase which is involved in the regulation of cell growth and expansion. This *H. glycines* CBM may thus be involved in syncytium expansion [19]. The CBMs present in *G. pallida* showed expression profiles reflecting these two functional roles; one CBM gene (GPLIN_000536400 – blue line in Figure 1D) is upregulated in J2 while another two CBM genes (GPLIN_000707900 and GPLIN_000706300 – green and red lines in Figure 1D) are upregulated at parasitic stages suggesting they could be the functional orthologues of the *H. glycines* genes involved in syncytium development (Figure 1D). Intriguingly, a similar pattern of expression was observed for the GH53 (arabinogalactan endo 1,4 β-galactosidase) genes; while one gene (GPLIN_000142900 – blue line in Figure 1E) was upregulated in J2 and particularly in males, expression of the other (GPLIN_000143000 – red line in Figure 1E) was restricted to the parasitic stages (Figure 1E). It was previously thought that nematode cell wall degrading enzymes are used solely for migration, with the plant’s own cell wall degrading machinery activated to allow the controlled modifications to the host cell wall that occur during syncytium formation [30, 31]. However, these expression data suggest that some nematode enzymes may also play a role in this process.

**Table 1:** **Genes potentially encoding cell wall modifying proteins in various nematode species**

Species/trophic life style	GH5	GH45	GH30	GH43	GH28	GH53	PL3	CBM	Expansin	Total
***Globodera pallida***	15	0	0	1	0	2	7	6	9	40
Sedentary endoparasitic PPN										
***Meloidogyne incognita***	21	0	6	2	2	0	30	9	20	90
Sedentary endoparasitic PPN										
***Meloidogyne hapla***	6	0	1	2	2	0	22	2	6	41
Sedentary endoparasitic PPN										
***Bursaphelenchus xylophilus***	0	11	0	0	0	0	15	0	8	34
Fungal feeder/facultative migratory endoparasite of trees										
***Caenorhabditis elegans***	0	0	0	0	0	0	0	0	0	0
Free-living bacterial feeder										
***Pristionchus pacificus***	6	0	0	0	0	0	0	0	0	6
Free-living bacterial feeder										
***Brugia malayi***	0	0	0	0	0	0	0	0	0	0
Animal parasite										

GH, Glycosyl hydrolase family; CBM, Carbohydrate binding module; PL, Pectate Lyase; PPN, plant-parasitic nematode.

### Orthologues of other previously characterised nematode effectors

The *G. pallida* predicted protein sequences were BLAST searched with a list of previously characterised effectors from the root-knot nematode *M. incognita* and the cyst nematodes *G. rostochiensis* and *H. glycines*. This analysis revealed 128 putative *G. pallida* orthologues of 37 effectors from these species (Additional file 1: Table S3). In 6 cases (*G. rostochiensis* sequences A42, 66P1 and 747, and *H. glycines* effectors Hgg6*,* G8H07 and G28B03), *G. pallida* orthologues were not present among the gene predictions from the final assembly but were present as uncalled sequences in the assembly and/or identified from transcriptome sequences. Several of these sequences were cloned from cDNA in order to ensure that they were genuinely present in *G. pallida* and for further functional analysis (below). In addition, 2 gene families similar to effectors IA7 and IVG9, previously identified from *G. pallida* were also present, containing 7 and 5 members respectively (Additional file 1: Table S3). A BLAST search of the *G. rostochiensis* assembled transcriptome (Eves van den Akker, unpublished) showed that, as expected, sequences similar to all of those identified in this analysis were present in *G. rostochiensis*. In addition to these sequences, two substantial gene families were present that could encode homologues of SPRYSEC effectors and orthologues of a sequence annotated as “*Heterodera avenae* gland cell protein” (Genbank HM147943.1)*.* These families have been described in detail as part of the *G. pallida* genome analysis and are not considered further here. Sixteen effectors from other cyst nematodes appeared to have no orthologue in the current assembly of the *G. pallida* genome or transcriptome (Additional file 2: Table S4). As previously described [27] almost no overlap (other than the cell wall modifying proteins and chorismate mutase) was found with effectors from *M. incognita*.

**Figure 2:**
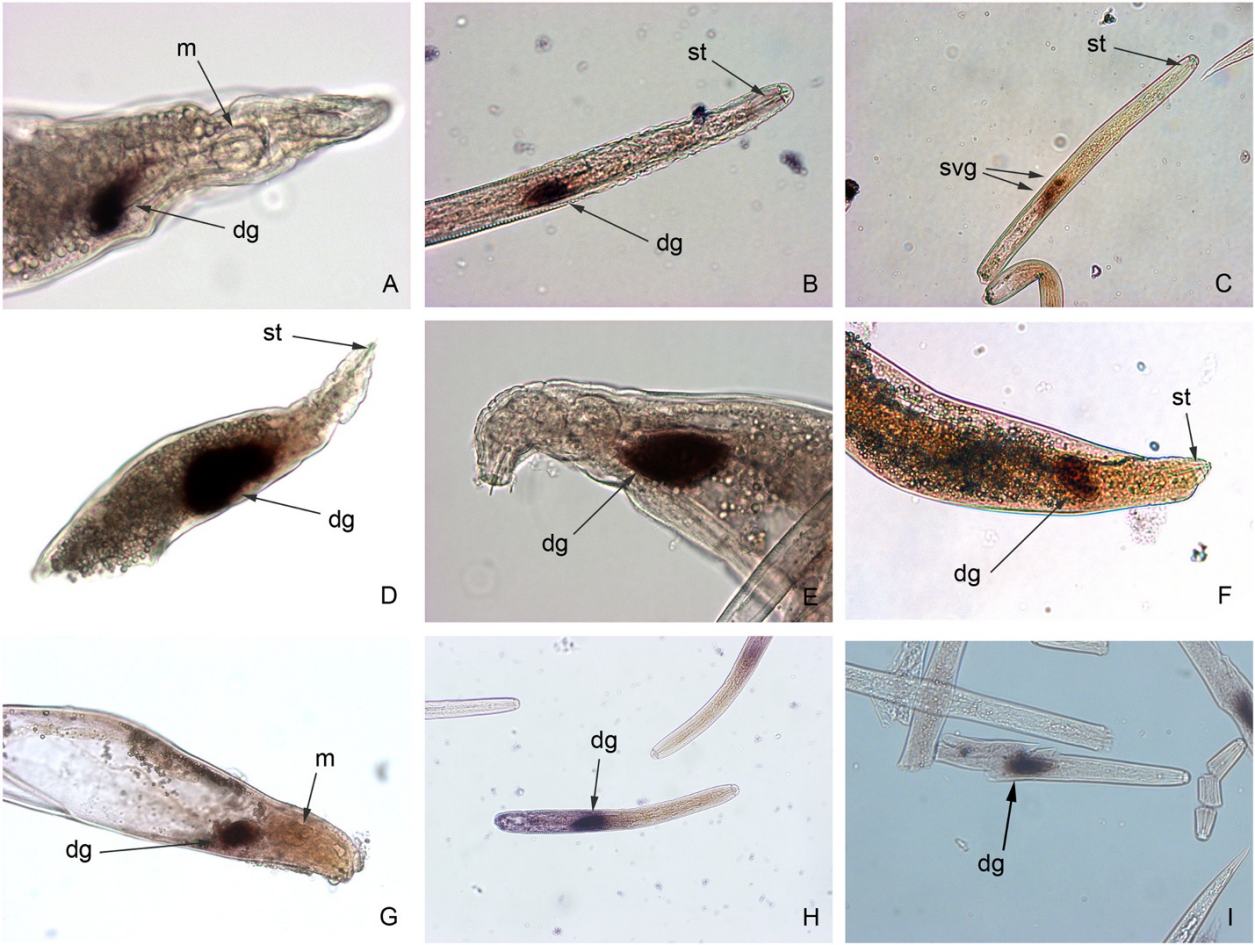
**Localisation of gene expression by** ***in situ*** **hybridisation of** ***Globodera pallida*** **SPRYSEC genes and orthologues of effectors from other PPN.** Sections of *G. pallida* preparasitic stage 2 juveniles **(B, C, E, H and I)** or 14dpi parasitic nematodes **(A, D, F and G)** were incubated with antisense probes based on the DNA coding sequence for the following gene loci: **(A)** GPLIN_001203000 (ortholog of *Heterodera glycines* 10C02 sequence); **(B)** GPLIN_000854400 (ortholog of *H. glycines* 16H02 sequence); **(C)** GPLIN_000662500 (ortholog of *H. glycines* G20E03 sequence); **(D)** GPLIN_000235400 (ortholog of *Globodera rostochiensis* 1106 sequence); **(E)** GPLIN_000243800 (ortholog of *H. glycines* 4D06 sequence); **(F)** *G. pallida* ortholog of *G. rostochiensis* A42 sequence; **(G)** GPLIN_000201400 (*G. pallida* ortholog of *G. rostochiensis* E9 sequence); **(H)** SPRYSEC GPLIN_001082900; **(I)** SPRYSEC GPLIN_000657200. st, stylet; dg, dorsal gland cell; svg, subventral gland cells; m, metacorpal bulb.

**Figure 3:**
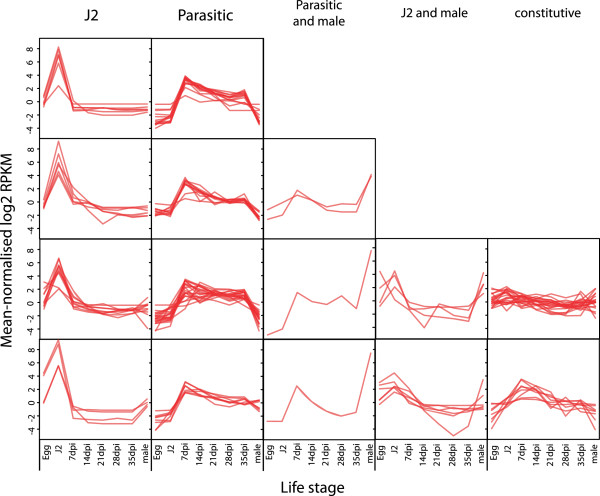
**Cluster analysis of co-regulated candidate effectors during** ***Globodera pallida*** **life cycle.** The y-axis represents fold change in expression values, determined by calculating fold changes over mean expression values across all samples from RNAseq data. Each cluster contains more than one sub group of genes with each sub group showing subtle differences in expression profile.

In order to demonstrate that the cyst nematode orthologues identified represent genuine effector candidates rather than secreted proteins with functions within the nematode body, we performed *in situ* hybridisation to confirm expression of selected genes in the gland cells of the nematode. This analysis identified genes expressed in the subventral gland cells and in the dorsal gland cell (Figure 2). Staining patterns in *G. pallida* matched those reported for the *H. glycines* orthologues, where these had previously been described ([10]; Figure 2A,C). In common with all other SPRYSEC sequences examined to date, the *G. pallida* SPRYSEC sequences tested were expressed in the dorsal gland cell (two examples shown in Figure 2H and I). In addition to confirming that the products of these genes represent genuine effector candidates, this analysis can provide information about the part of the life cycle in which the effectors are important as the subventral gland cells are thought to produce proteins required at the early stages of the parasitic process while the dorsal gland cells produce proteins that are important at later stages. On a larger scale, we were able to undertake a cluster analysis of the temporal expression profiles of all effector candidates using the RNAseq data generated for *G. pallida*. This analysis revealed that five different clusters of genes sharing similar expression profiles were present: J2 (30 sequences), J2 and male (5 sequences), parasitic stages (61 sequences), constitutive (20 sequences) and parasitic and male (4 sequences) (Figure 3). Comparing the *in situ* hybridisation data with this cluster information gave conflicting results. All the genes expressed in the subventral gland cells showed expression peaks at J2 (*e.g.* GPLIN_000662500 and many of the cell wall degrading enzymes), suggesting that expression in these tissues is usually indicative of a role at the earliest stages of the host-parasite interaction. However, genes expressed in the dorsal gland cell showed a variety of different temporal expression profiles with some showing a clear peak of expression at the J2 stage while others peaked in expression during the parasitic stages as expected. This implies that control of the temporal expression of effectors is complex and is not, as previously suggested, simply a question of expression in one gland cell or another.

### *G. pallida*effectors target various host subcellular structures

Understanding the subcellular localisation of effectors can provide information about their putative function(s), can be used to prioritise future functional studies and is also of value when analysing the results of yeast two-hybrid screens that aim to identify targets of effectors. Effectors from a range of plant pathogens have been shown to target diverse subcellular structures in plant cells. Several studies have shown in particular that some effectors from either cyst or root-knot nematodes can target the host cell nucleus and/or nucleolus, suggesting that PPN may be hijacking key nuclear functions in their host [8, 32–34].

**Figure 4:**
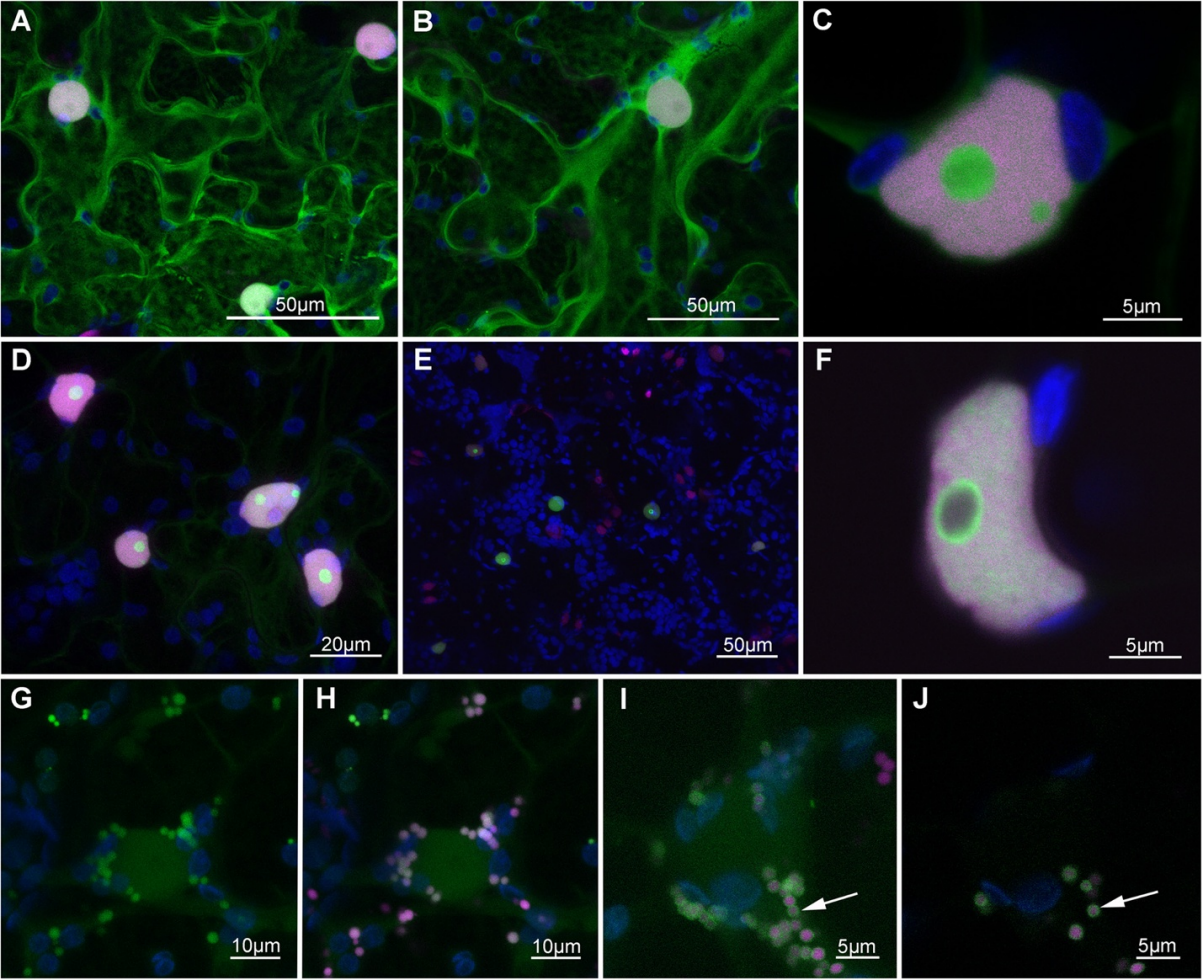
**Subcellular localisations of** ***Globodera pallida*** **effector fusions with the enhanced green fluorescent protein (eGFP) in leaves of** ***Nicotiana benthamiana*** **. (A)** Localisation of free eGFP*.* **(B)** Localisation of GPLIN_000015300 (ortholog of *Heterodera glycines* G7E05 sequence) in the cytoplasm and nucleoplasm but excluded from the nucleolus. **(C)** Localisation of GpA42 _ (ortholog of *Globodera rostochiensis* A42 sequence) in the nucleoli. **(D)** Faint expression of GPLIN_000235400 (ortholog of *G. rostochiensis* 1106 sequence) in the cytoplasm with accumulation in the nucleus, particularly in the nucleoli. **(E & F)** Exclusion of SPRYSEC effector GPLIN_001465500 from the cytoplasm but accumulation in the nucleoplasm, particularly surrounding the nucleolus. **(G-H)** Expression of GPLIN_000662500 (ortholog of *H. glycines* G20E03 sequence) in peroxisomes. **(I-J)** Expression of GPLIN_000457000 (ortholog of *H. glycines* hgsec4 sequence) in the peroxisome membrane (arrows). In all images the eGFP-effector fusion is seen in green, with monomeric-red fluorescent protein (mRFP) co-label in magenta, from either transgenic expression of an mRFP-histone fusion **(A-F)** or co-expression of an mRFP-tagged peroxisome marker **(G-J)**, and autofluorescent chloroplasts displayed in blue. Scale bars represent 50 μm for **A**, **D**, 20 μm for **C**, 10 μm for **G**, **H**, and 5 μm for **E**, **F**, **I** and **J**. Localisation of *Agrobacterium*-mediated transient expression of eGFP-effector fusions was observed 48 h post inoculation by confocal microscopy.

Using eGFP-fusions in transient expression assays in *Nicotiana benthamiana*, we observed that putative effectors from *G. pallida* targeted a wide range of plant cell structures. While many were localised throughout the cytoplasm in a manner similar to free eGFP (Figure 4A and B), others showed more restricted localisation. Although many effectors are small enough to allow passive diffusion into the nucleus as eGFP fusions (*e.g.* Figure 4B), these are excluded from the nucleolus. However, several effectors were identified that were localised in the nucleolus (Figure 4C and D) and these are likely to represent genuine localisations rather than passive diffusion. These two effectors have nuclear localisation signals (NLS) predicted by PSORT. One SPRYSEC effector (GPLIN_001465500) looked to be excluded from the cytoplasm but was localised specifically in the nucleus and was often seen surrounding the nucleolus (Figure 4E and F). No NLS was predicted for this effector sequence. Similar localisations have been observed for some viral proteins [35]. Effectors were also identified that targeted peroxisomes and the peroxisome membrane (Figure 4G-I). This localisation was confirmed using an mRFP-tagged marker for peroxisomes.

*Globodera pallida* induces profound gene expression changes in its host when establishing a feeding site. It is possible that effectors which target the nuclei may manipulate gene expression directly either as a part of the process of establishing the feeding site or in order to suppress defence signalling pathways. Alternatively, such effectors may act indirectly, targeting host transcription factors or other nuclear proteins, which may interfere with nuclear functions. Effectors from a range of cyst [32] and root-knot nematodes [33] that localise to the nucleus have been identified, demonstrating the importance of targeting this structure for a range of biotrophic PPN groups (reviewed in [36]), as is the case for other plant pathogens. By contrast, this is the first report of any nematode effector that targets the peroxisome. Indeed, no direct evidence for targeting of this organelle by any plant pathogen has been reported previously, although a peroxisome targeting signal has been identified in the DspA/E effector of *Erwinia amylovora* [37]. Plant peroxisomes are involved in a variety of metabolic processes, including responses to abiotic stress and production of auxins [38], both of which are clearly of relevance to the *G. pallida* life cycle. For example, part of the biosynthetic pathway for jasmonic acid occurs in the peroxisomes and a mutant line lacking one of the enzymes involved in this process in *Solanum lycopersicum* is impaired in its ability to mount defence responses against some insects [39]. The peroxisome is also an important site for production of hydrogen peroxide [40], which is known to be deployed as part of the defence response against PPN [41]. Less is known about the nature and roles of proteins present in the peroxisomal membrane, although several are known to control the import/export of other proteins and metabolites into/from the peroxisome (reviewed in [42]. Information on the effectors from *G. pallida* that target the peroxisomes will be useful in terms of framing future functional studies.

### Novel effector candidates

**Figure 5:**
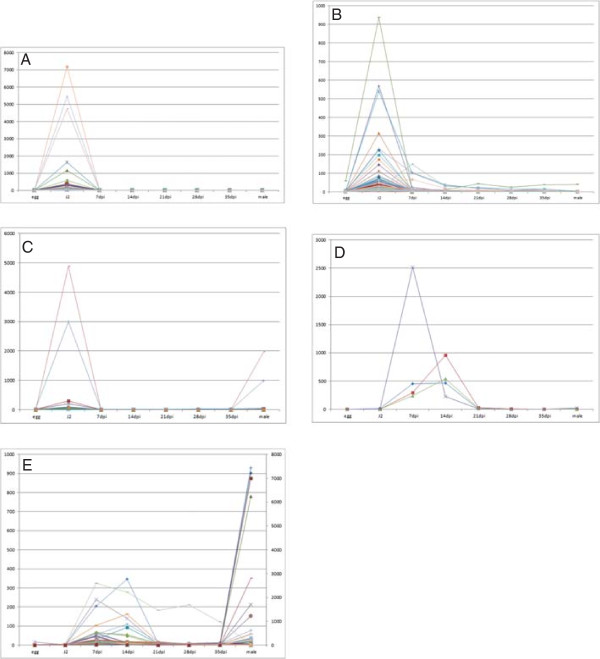
**Expression profiles of “novel” effector candidates across the** ***G. pallida*** **life cycle.** The Y-axis represents reads per kilobase per million. Clusters represent: **(A)** J2 specific; **(B)** J2 and parasitic; **(C)** J2 and male; **(D)** parasitic only; **(E)** parasitic and male.

The analysis performed as part of the *G. pallida* genome project [27], identified 117 potential novel effectors (Additional file 3: Table S5). These predicted proteins have a signal peptide, lack a transmembrane domain and were upregulated at J2 (vs egg) or at early parasitic stages (vs J2). We have subsequently performed further analysis of these 117 proteins. We first examined the expression profiles of each of the candidates across the *G. pallida* life cycle as only the expression at two life stages was considered during the identification process. This analysis showed that, as for the orthologues of effectors from other species (above), the expression profiles formed five clusters (Figure 5): J2 only (28 sequences), J2 and parasitic (46 sequences), J2 and male (8 sequences), parasitic (4 sequences) and parasitic and male (31 sequences). The candidate effectors were also analysed for the presence of Pfam domains. Twenty seven potential Pfam domains were identified for 11 of the 117 predicted proteins (Table 2) while the other 106 sequences contained no known domains and, since these also have no BLAST matches against NR using the stringency requirements described here, they therefore represent novel proteins. A BLAST search of assembled *G. rostochiensis* transcripts showed that all but two of the *G. pallida* novel effector candidates had strong matches in *G. rostochiensis* (not shown). Since the transcriptome assembly (generated from J2s and parasitic *G. rostochiensis* 14 days after infection) is likely to be incomplete, compared to the full *G. pallida* genome, it is reasonable to conclude that the “novel” effector candidates do not include any *G. pallida* specific sequences. By contrast, only 17 of the sequences had matches in the *M. incognita* proteins predicted from the genome sequence, suggesting that the dataset is enriched in proteins specific to cyst nematodes.

**Table 2:** **PFam analysis of “novel” candidate effectors**

Gene	Pfam domain	Accession	CDS length	Domain length	E-value
GPLIN_000948600	EF_hand_3	PF13202.1	137	25	9.30E-15
GPLIN_000948600	EF_hand_3	PF13202.1	137	25	9.30E-15
GPLIN_000948600	EF_hand_4	PF13405.1	137	31	8.30E-10
GPLIN_000948600	EF_hand_4	PF13405.1	137	31	8.30E-10
GPLIN_000948600	EF_hand_6	PF13833.1	137	54	1.60E-10
GPLIN_000948600	EF_hand_6	PF13833.1	137	54	1.60E-10
GPLIN_000776900	Gal-bind_lectin	PF00337.17	926	133	2.50E-13
GPLIN_000208700	Homeobox	PF00046.24	164	57	2.30E-08
GPLIN_000510600	Pkinase	PF00069.20	320	260	4.20E-57
GPLIN_001391000	Pkinase	PF00069.20	374	260	1.70E-08
GPLIN_000510600	Pkinase_Tyr	PF07714.12	320	259	7.20E-32
GPLIN_001318000	UQ_con	PF00179.21	182	140	2.70E-42
GPLIN_001268500	UQ_con	PF00179.21	305	140	1.50E-30
GPLIN_000075700	VWA	PF00092.23	195	179	4.70E-09
GPLIN_000075700	VWA_2	PF13519.1	195	172	1.50E-10
GPLIN_000713500	zf-C2H2	PF00096.21	161	23	4.30E-13
GPLIN_000713500	zf-C2H2	PF00096.21	161	23	4.30E-13
GPLIN_000713500	zf-C2H2	PF00096.21	161	23	4.30E-13
GPLIN_000713500	zf-C2H2_4	PF13894.1	161	24	2.90E-10
GPLIN_000713500	zf-C2H2_4	PF13894.1	161	24	2.90E-10
GPLIN_000713500	zf-C2H2_4	PF13894.1	161	24	2.90E-10
GPLIN_000589200	zf-C3HC4_3	PF13920.1	544	50	2.30E-13
GPLIN_000713500	zf-H2C2_2	PF13465.1	161	26	2.50E-16
GPLIN_000713500	zf-H2C2_2	PF13465.1	161	26	2.50E-16
GPLIN_000713500	zf-H2C2_2	PF13465.1	161	26	2.50E-16
GPLIN_000271900	zf-rbx1	PF12678.2	297	75	8.00E-10
GPLIN_000271900	zf-RING_2	PF13639.1	297	46	2.50E-11

The identified Pfam domains are: EF_hand: a helix-loop-helix domain that is thought to be involved in calcium binding; Gal-bind_lectin: lectin domain thought to be involved in binding β-galactoside; Homeobox: homeobox transcription factors; Pkinase: protein kinase domains; UQ_con: ubiquitin E2 conjugating domains; VWA: von Willebrand factor domain; zf: zinc finger domain; zf-rbx1: zinc finger domain; zf-RING_2: domain contains a zinc finger and a domain associated with E3 ligase activity. Duplicate entries indicate that the domain is present more than once in the identified protein.

**Figure 6:**
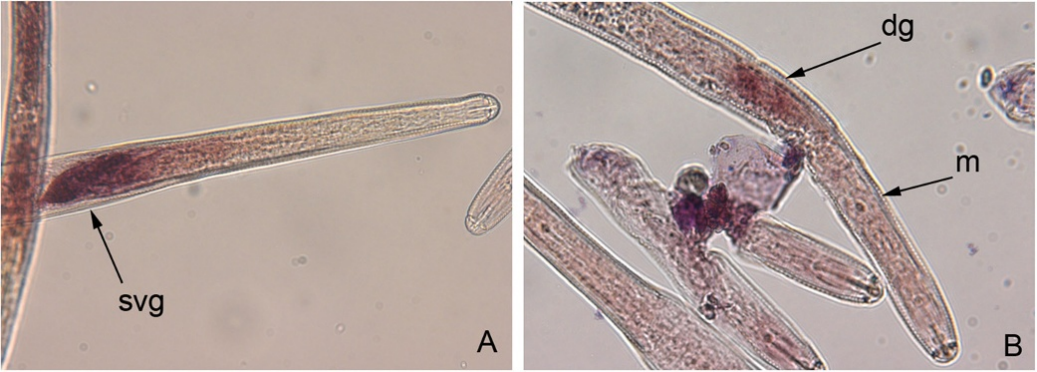
**Localisation of “novel”** ***G. pallida*** **candidate effectors expression by** ***in situ*** **hybridisation to preparasitic second stage juveniles (J2s).** Sections of nematodes were incubated with antisense probes designed based on DNA coding sequence for the following gene loci **(A)** GPLIN_000333000; **(B)** GPLIN_000834600; dg, dorsal gland cell; m, metacorpal bulb; svg, subventral gland cell.

In order to confirm that at least some of the novel effector candidates represented genuine effectors we performed *in situ* hybridisation to determine the site of expression of some of the candidates. *In situ* hybridisation with nematodes is a labour intensive process meaning that it was not feasible to analyse most of the sequences. However, two of the “novel” candidate effectors that were upregulated at J2 (GPLIN_000333000 and GPLIN_000834600) were expressed in the subventral and dorsal gland cells respectively (Figure 6A and B). GPLIN_000333000 is part of a small family (~7 sequences) of related proteins, each of which has a signal peptide. These data therefore suggest that it represents a novel effector gene family in *G. pallida.*

### Genomic organisation of effectors and diversification by alternative splicing

Several of the *G. pallida* effector gene families are considerably expanded and this may be a reflection of selection pressure to avoid detection or maintain function within a host that is under selection pressure to evade infection. For example, one *G. pallida* effector gene family, similar to previously identified effectors from *H. glycines* (4D06 and several other related sequences) consists of 39 members in *G. pallida*. However, the most striking examples of expansion are found in the SPRY domain proteins and a family of proteins similar to a sequence annotated as “*H. avenae* dorsal gland protein” in the database. These families consist of 299 and 295 members respectively. We analysed clustering of these sequences in the *G. pallida* genome and found that 180/295 of the sequences similar to “*H. avenae* dorsal gland protein” are clustered (*i.e.* present on the same *G. pallida* scaffold) with the largest cluster consisting of 10 similar genes in a group of 14 genes on scaffold 299 (Additional file 4: Figure S1). Similarly, 152/299 of the SPRY domain proteins are located in clusters present on individual scaffolds, with the largest cluster consisting of 7 genes on scaffold 141 (Additional file 4: Figure S1). These estimates of clustering are likely to be significant underestimates, given the fragmented nature of the *G. pallida* genome sequence. These data suggest that the SPRY domain and “dorsal gland protein” gene families are likely to have expanded, at least in part, by a process of tandem duplication followed by diversification.

It has recently been shown that for several plant pathogens, including the late blight pathogen *Phytophthora infestans,* effector sequences are more likely to be found in repeat-rich, gene sparse regions of the genome [43]. This property has been used to identify novel candidate virulence factors [44]. Calculating the distance between each gene and its nearest neighbours (the flanking intergenic regions) and comparing these values for all genes in a genome, can be used as a way of calculating whether a gene resides in a gene rich or gene sparse environment [45]. We therefore examined whether or not this pattern is also true in the *G. pallida* genome with a particular focus on the SPRYSECs as the nature of this substantial gene family and the absence of a similarly expanded family in other cyst nematodes suggested that it may be in the process of rapid evolution. Although this type of analysis is challenging for genomes as fragmented as that of *G. pallida*, with two data points being lost at the end of each contig, the pattern reported for other plant pathogens did not seem to be followed in *G. pallida*, with the distribution patterns of the effectors not significantly different from those of other genes (Additional file 5: Figure S2).

Alternative splicing generates multiple mRNA transcripts from a single precursor and can lead to changes in RNA or protein levels, or can give rise to different forms of related proteins from a single gene. These may differ in terms of function or cellular localisation (reviewed in [46]). In addition, effectors are in direct contact with the host and are therefore likely to be under strong diversifying selection pressure in order to produce variants that evade detection. Alternative splicing is one mechanism by which this may be achieved and has been described previously for one cyst nematode effector [47]. We analysed the *G. pallida* effectors similar to those from other nematode species (orthologues of other previously characterised nematode effectors as described above) for evidence of alternative splicing. Mapping of transcripts to the predicted genome sequence for these genes indicated that at least 38% undergo alternative splicing. Some effector gene families showed evidence for more frequent alternative splicing: Nineteen of 39 members of the *G. pallida* gene family similar to the *H. glycines* “4D06” genes and 3 of 4 *G. pallida* orthologues of the *H. glycines* G20E03 sequence showed evidence for alternative splicing. Evidence was obtained for alternative splicing occurring within a single life stage (Additional file 6: Figure S3A). Intriguingly, evidence supporting the generation of different mRNAs at different life stages was also obtained (Additional file 6: Figure S3B). Although functional studies are required in order to assess the relevance of this observation, this raises the possibility that alternative splicing generates forms of a protein with different functional properties at different life stages, or that this may be another mechanism by which effector activity is regulated. In addition, these data suggest that alternative splicing may be relatively widespread within effector sequences of cyst nematodes.

The data presented here demonstrate that *G. pallida* deploys a significant number of effectors in order to promote its biotrophic interactions with the host. These effectors are under precise temporal regulation and target a variety of host subcellular structures and, most likely, a variety of host processes. The availability of these sequences will greatly facilitate future functional studies on this, and related cyst nematodes and enormously expand the range of potential targets for novel control strategies based, for example, on RNA-interference [48]. A proof of concept study targeting an effector of root-knot nematode [49] demonstrated the feasibility of such approach in the model plant *Arabidopsis*, where it conferred broad resistance to several *Meloidogyne* species.

## Conclusions

- The genome of *G. pallida* has several hundred genes that could encode putative effectors. Further diversity in effectors is generated through alternative splicing. In the present *G. pallida* assembly there was no evidence to suggest that effectors are preferentially localised in gene sparse regions of the genome.
- A bioinformatic pipeline combining analysis of the presence of a signal peptide, absence of a transmembrane domain and expression profiling can be used to identify novel effectors.
- Effectors display distinct temporal expression profiles across the *G. pallida* life cycle, suggesting that different effectors are deployed in order to support different phases of the parasitic process.
- Effectors of *G. pallida* target a range of host structures including the nucleus and peroxisomes.

## Methods

### *G. pallida*resources & alternative splicing analysis

The biological material used for both genome and transcriptome analysis originate from a standard Pa2/3 pathotype of *G. pallida* population “Lindley” which is held at the James Hutton Institute, Dundee, UK [50]. The *G. pallida* predicted protein set version 1.0 (16th May 2012) was used for identification of effectors. This protein set is available at ftp://ftp.sanger.ac.uk/pub/pathogens/Globodera/pallida/ and was used for the detailed analysis of the *G. pallida* genome [27]. Expression profiles of effectors across the life cycle were determined analysing the RNAseq information available for *G. pallida* [27]; replicated RNAseq datasets from eggs (containing unhatched J2), invasive stage J2, parasitic nematodes at 7, 14, 21, 28 and 35 days post infection (dpi) and adult males. Distribution of effectors in gene sparse versus gene rich regions was analysed as described in [45]. Scaffold drawings indicating the direction and location of genes were produced using a combination of Biopython [51] and Genome Diagram [52].

For analysis of alternative splicing, genomic regions of the effectors present in other nematode species (referred to as “orthologue effectors” below) were first extracted from the genome sequence and compared to the orthologues using BLAT. Life stage specific *de novo* transcriptome assemblies for the J2 and 7dpi RNAseq data were generated using Trinity as previously described [53] and compared to the genomic regions using BLAT with 90% similarity cut off. All tracks were visualised in IGV (Integrative Genome Viewer [54]. Potential alternative splicing events, where more than one transcript mapped to the same genomic copy, were checked manually to assess the impact of putative alternative splice events.

### Identification of *G. pallida*orthologues of previously characterised effectors

A list of known effectors from other PPN was collated using data from *H. glycines* gland cell ESTs [10, 55], microarray analysis [56], effectors identified from cDNA-AFLP analysis on *G. rostochiensis* [9], *G. rostochiensis* and *G. pallida* ESTs [57] and effectors identified from *M. incognita* [58]. The list also included effectors that had previously been identified from *G. pallida* [59, 60]. In addition, a list of *G. rostochiensis* effectors was provided by Dr. G. Smant (Wageningen University, Netherlands). The collated effector list, consisting of 133 *G. rostochiensis* sequences, 53 *H. glycines* sequences, three *G. pallida* sequences and 35 *M. incognita* sequences, was subjected to a local, command line BLAST [61] against the *G. pallida* genome sequence. This search used an E-value threshold of 10^−5^ with low complexity filtering turned off.

### CAZymes and other cell wall modifying proteins

The CAZymes Analysis Toolkit (CAT) [62]; was used to identify putative carbohydrate active enzymes (CAZymes) with a predefined CAZyme database on the *G. pallida* predicted protein set. Putative CAZymes were manually annotated using a combination of BLASTP Vs NR database, NCBI’s Conserved Domain Database service [63] and InterProScan [64] to determine to presence of the catalytic domains. Genes of interest were identified by parsing the CAT output files. Databases of expansins and carbohydrate binding module (CBM) genes from other PPN were used for BLASTP searches against the *G. pallida* genome*.*

### Identification of novel candidate effectors

The predicted *G. pallida* protein set was first analysed using a standard secretory protein identification protocol. Proteins that had a predicted signal peptide and no transmembrane domain were identified using SignalP 3.0 [65] followed by TMHMM [66], based on the methodology used in [8] using the Galaxy tools and workflow described in [67]. Expression profiles of the genes that passed these filters were then analysed using DESeq (http://bioconductor.org/packages/release/bioc/html/DESeq.html) [68] in order to identify genes that were significantly upregulated in J2 compared to eggs or upregulated at 7 dpi compared to J2. Genes that passed this expression profiling filter were then BLAST searched against the NR database and those that obviously had functions unrelated to parasitism (*e.g.* collagens, digestive proteinases) were manually removed. In some cases the results of this BLAST searching provided functional information about the novel putative effectors. The putative effector list was thus analysed for any known domains using Pfam rules defined in ftp://ftp.sanger.ac.uk/pub/databases/Pfam/current_release/Pfam-A.hmm.gz (July 2012), using HMMER [69]. All sequences passing these filters were then BLAST searched against the proteins predicted in the *M. incognita* genome sequence [21] and against an assembled transcriptome derived from an RNAseq dataset of *G. rostochiensis* J2 and parasitic nematodes at 14 dpi (S. Eves van den Akker, unpublished). The sequences were also BLAST searched against dbEST in order to identify matches in other plant parasitic nematodes.

### Analysis of effector expression profiles

The expression profiles of putative effectors were analysed using the normalised RNAseq data generated as part of the *G. pallida* genome project. The MBClusterseq program (http://cran.r-project.org/web/packages/MBCluster.Seq/index.html) was used to separate the effectors with similar expression profiles into clusters. Inspection of the results of this analysis revealed that some clusters showed very similar patterns and genes in such clusters were subsequently merged into the same cluster.

### *In situ*hybridisation

The spatial expression patterns of some candidate effectors were examined by *in situ* hybridisation as previously described [60]. The sequences of primers used to amplify fragments of these genes for probe synthesis are provided in Additional file 7: Table S1.

### Cloning and characterisation of effectors

Messenger RNAs were isolated from J2 or parasitic stage *G. pallida* (population Lindley) using a Dynabeads mRNA Direct Micro kit (Invitrogen) and treated with RQ1 DNase (Promega). cDNA was synthesised from approximately 400 ng purified mRNA using the Superscript III system (Invitrogen) with poly(dT) primers following the manufacturer’s instructions. For cloning, the coding sequences of selected effector candidates were amplified by PCR from cDNA, excluding the predicted signal peptide sequence but with the ACCATG leader sequence and a stop codon in the forward and reverse primer respectively (Additional file 8: Table S2). PCR was performed using the proof reading KOD DNA polymerase (Novagen) and products were resolved on 1.5% (w/v) agarose gels. Amplification products of the expected size were purified from gels using the QIAquick Gel Extraction Kit (QIAGEN) and inserted into the pCR8/GW/TOPO Gateway ENTRY vector by TA cloning following the manufacturer’s instructions (Invitrogen). Clones were subsequently recombined into the binary pK7WGF2 expression vector [70] for fusion with the enhanced green fluorescent protein (eGFP) tag using LR clonase (Invitrogen) following the manufacturer’s instructions. The integrity of the effector sequence in both ENTRY clones and in the destination vectors, as well as the fusion with the eGFP were confirmed by sequencing. For *Agrobacterium*-mediated transient expression assays, the eGFP-fusion expression vectors (Spectinomycin selection) were transferred by electroporation to *Agrobacterium tumefaciens* strain GV3101 that contains a helper vector encoding *virG*^N54D^ (Gentamycin selection) [71].

### Transient expression and analysis of subcellular localisation *in planta*

For subcellular localisation of the eGFP-effector fusions *in planta*, the constructs were transiently expressed in leaves of 4-week-old *Nicotiana benthamiana* using *Agrobacterium*-mediated transformation. *Agrobacterium* clones were grown overnight at 28°C in 5 mL Luria Bertani (LB) medium containing 25 μg/L Gentamycin and 100 μg/mL Spectinomycin. Bacterial cells were pelleted by centrifugation, rinsed and resuspended in infiltration buffer containing 10 mM MgCl_2_, 10 mM MES (2-[*N*-Morpholino] ethane sulfonic acid), and 200 μM acetosyringone, and adjusted to an optical density at 600 nm (OD_600nm_) of 1. Bacteria were then incubated for at least 3 h in the dark at room temperature prior to further dilution in infiltration buffer to OD_600nm_ of 0.02 per construct and infiltration on the abaxial side of the leaves using a 1-mL needleless syringe.

For co-localisation analysis, bacteria were either infiltrated in leaves of transgenic *N. benthamiana* line (CB157) expressing a nuclear histone marker fused to mRFP (mRFP-H2B) [72] or co-infiltrated into wild-type plants together with an *A. tumefaciens* clone containing a peroxisome marker fused to mRFP (PfluB4; 50 μg/mL Kanamycin selection) [73]. Localisations were imaged 48 h post inoculation using either a Zeiss LSM 710 or a Leica SP2 confocal laser-scanning microscope. eGFP was imaged with an excitation wavelength (λ) of 488 nm and emission at λ495-530 nm (λ505-530 nm for SP2). Autofluorescence from chlorophyll generated by excitation at this wavelength was collected at λ657-737 nm (SP2 λ650-700 nm). mRFP was imaged sequentially with an excitation at λ561 nm and emission at λ592-632 nm (SP2 λ580-610 nm). Western blots were performed using standard protocols to analyse the size of expressed proteins – in all cases where a signal was detected the size of the band was in agreement with the predicted size of the eGFP-effector fusion protein with no evidence for degradation (not shown).

## Availability of supporting data

Sequence data analysed in this paper were part of the *G. pallida* genome project. All data generated in this project have been submitted to the Genbank database under the accession number PRJEB123. Data and annotation have been submitted to Wormbase and are available at http://parasite.wormbase.org/Globodera_pallida_prjeb123/Info/Index. The *G. pallida* genome assembly and functional annotation is available from ftp://ftp.sanger.ac.uk/pub/project/pathogens/Globodera/pallida and via GeneDB at http://www.genedb.org/Homepage/Gpallida.

## Electronic supplementary material

Additional file 1: Table S3: *G. pallida* genes similar to effectors characterised from other nematodes (excluding cell wall degrading and modifying enzymes). Expression profiles of each gene as inferred from RNAseq analysis are indicated. (DOCX 23 KB)

Additional file 2: Table S4: Previously identified effectors from other cyst nematodes absent from the current *G. pallida* genome assembly. (DOCX 15 KB)

Additional file 3: Table S5: *Globodera pallida* secreted proteins up-regulated in J2 or early parasitic stages that may represent novel effector candidates. (DOCX 16 KB)

Additional file 4: Figure S1: Locations of exons (coloured arrows) in scaffolds 299 **(A)** and 141 **(B)**. Exons of genes encoding proteins similar to “*H. avenae* dorsal gland cell protein” are indicated in purple on panel A and exons of genes encoding proteins that contain a SPRY domain are indicated in yellow on panel B. Other predicted genes are indicated in blue or green (alternating genes). Direction of arrows indicates orientation of predicted open reading frames. Grey shading indicates unsequenced regions of scaffolds. (PNG 495 KB)

Additional file 5: Figure S2: Distribution of various effector groups across gene sparse and gene rich regions of the *Globodera pallida* genome. Heat map generated reflecting gene density and the distribution of three classes of effector candidates: the SPRYSECs, *G. pallida* orthologs of effectors from other PPN and “novel” effectors. (PNG 12 KB)

Additional file 6: Figure S3: Example of potential alternative splicing events in *G. pallida* effectors **Figure S3A:** Alternative splicing of GPLIN_000359000 within one life stage. Black bar indicates predicted sequence from genome with bars showing predicted coding region from gene model; introns are shown as lines. Red bars indicate *de novo* assembled transcripts from RNA extracted from parasitic nematodes 7dpi, red lines indicate gaps compared to genome sequence. **Figure S3B:** Alternative splicing of GPLIN_000243800 between life stages. Black bar indicates predicted sequence from genome with bars showing predicted coding region from gene model; introns are shown as lines. Red and blue bars/lines indicate *de novo* assembled transcripts from parasitic nematodes 7dpi and J2s respectively. (TIFF 2 MB)

Additional file 7: Table S1: Primers used to generate DNA fragments used for synthesis of probes for *in situ* hybridisation. (DOCX 14 KB)

Additional file 8: Table S2: Sequences of primers used for cloning full length effectors (without signal peptides). (DOCX 14 KB)
