# Supplementary material for: Genomic characterisation of the effector complement of the potato cyst nematode Globodera pallida
Source: BMC Genomics. 2014 Oct 23;15(1):923. doi: 10.1186/1471-2164-15-923 (PMC4213498; doi:10.1186/1471-2164-15-923)
Supplement: Supplementary file 1 — Additional file 1: Table S3: G. pallida genes similar to effectors characterised from other nematodes (excluding cell wall degrading and modifying enzymes). Expression profiles of each gene as inferred from RNAseq analysis are indicated. (DOCX 23 KB) [file 12864_2014_6605_MOESM1_ESM.docx]

| ***G. pallida* gene** | **Life-stage expression** | **Effector names** |
| --- | --- | --- |
| GPLIN_000594000 | J2 | C52 effector protein-like |
| GPLIN_000376700 | J2 | Chorismate mutase effector |
| GPLIN_000780600 | J2 | *G. pallida*  homologue of *H. glycines* effector G19C07 |
| GPLIN_000854400 | J2 | *G. pallida*  homologue of *H. glycines* G16H02 effector |
| GPLIN_000638300 | J2 | *G. pallida*  IA7 effector |
| GPLIN_000591100 | J2 | *G. pallida*  IVG9 effector |
| GPLIN_000167700 | J2 | GpUBI-EP effector similar to Ubiquitin extension proteins |
| GPLIN_000407400 | J2 | Member of effector gene family similar to *H. glycines* effector 4D06 |
| GPLIN_001456900 | J2 | Member of effector gene family similar to *H. glycines* effector 4D06 |
| GPLIN_000207700 | J2 | Member of the d gl-1 effector family |
| GPLIN_000325200 | J2 | Member of the d gl-1 effector family |
| GPLIN_000901700 | J2 | Member of the d gl-1 effector family |
| GPLIN_001317500 | J2 | Member of the d gl-1 effector family |
| GPLIN_000740500 | J2 | Paralogue of IA7 effector |
| GPLIN_000293500 | J2 | Paralogue of IVG9 effector |
| GPLIN_001541500 | J2 | Paralogue of IVG9 effector |
| GPLIN_001098200 | J2 | Possible paralogue of IVG9 effector |
| GPLIN_001110200 | J2 | Possible paralogue of IVG9 effector |
| GPLIN_000057600 | J2 | Similar to *G. rostochiensis* E9 effector protein |
| GPLIN_000187800 | J2 | Similar to *G. rostochiensis* E9 effector protein |
| GPLIN_000201400 | J2 | Similar to *G. rostochiensis* E9 effector protein |
| GPLIN_000872800 | J2 | Similar to *H. glycines* effector 33A09 |
| GPLIN_000996800 | J2 | Similar to *H. glycines* effector G12H04 |
| GPLIN_000297600 | J2 | Similar to *H. glycines* effector G17G01 |
| GPLIN_001526900 | J2 | Similar to *H. glycines* effector G17G01 |
| GPLIN_000662500 | J2 | Similar to *H. glycines* G20E03 effector |
| GPLIN_000926600 | J2 | Similar to *H. glycines* G20E03 effector |
| GPLIN_000962200 | J2 | Similar to *H. glycines* G20E03 effector |
| GPLIN_000977100 | J2 | Similar to *H. glycines* G20E03 effector |
| GPLIN_000555600 | J2 | Similar to *M. incognita*  effector AY135365, J2 specific |
| GPLIN_000604400 | J2 | Similar to *M. incognita*  effector AY135365, J2 specific |
| GPLIN_001199500 | J2 and Male | Member of the d gl-1 effector family |
| GPLIN_000178900 | J2 and Male | Similar to *H. glycines* secretory protein 11 putative effector. Similar to transthyretin-like proteins |
| GPLIN_000738800 | J2 and Male | Similar to *H. glycines* secretory protein 11 putative effector. Similar to transthyretin-like proteins |
| GPLIN_001559100 | J2 and Male | Similar to *H. glycines* secretory protein 11 putative effector. Similar to transthyretin-like proteins |
| GPLIN_001475500 | J2 and Male | Similar to RKN effector (gland cell protein 28). Similar to other nematode proteins |
| GPLIN_000666500 | Parasitic | Chorismate mutase effector |
| GPLIN_000668700 | Parasitic | *G. pallida*  homologue of *H. glycines* effectors 25A01 and 30G12 |
| GPLIN_000668700 | Parasitic | Similar to *H. glycines* 30G12 effector |
| GPLIN_000642100 | Parasitic | Effector similar to Ubiquitin extension proteins |
| GPLIN_000119200 | Parasitic | Member of 1106 effector gene family |
| GPLIN_000235400 | Parasitic | Member of 1106 effector gene family |
| GPLIN_000314000 | Parasitic | Member of 1106 effector gene family |
| GPLIN_000359000 | Parasitic | Member of 1106 effector gene family |
| GPLIN_000683800 | Parasitic | Member of 1106 effector gene family |
| GPLIN_000684200 | Parasitic | Member of 1106 effector gene family |
| GPLIN_000768400 | Parasitic | Member of 1106 effector gene family |
| GPLIN_000793000 | Parasitic | Member of 1106 effector gene family |
| GPLIN_000850500 | Parasitic | Member of 1106 effector gene family |
| GPLIN_001295300 | Parasitic | Member of 1106 effector gene family |
| GPLIN_001613000 | Parasitic | Member of 1106 effector gene family |
| GPLIN_000060800 | Parasitic | Member of effector gene family similar to *H. glycines* effector 4D06 |
| GPLIN_000072400 | Parasitic | Member of effector gene family similar to *H. glycines* effector 4D06 |
| GPLIN_000126500 | Parasitic | Member of effector gene family similar to *H. glycines* effector 4D06 |
| GPLIN_000203300 | Parasitic | Member of effector gene family similar to *H. glycines* effector 4D06 |
| GPLIN_000243700 | Parasitic | Member of effector gene family similar to *H. glycines* effector 4D06 |
| GPLIN_000243800 | Parasitic | Member of effector gene family similar to *H. glycines* effector 4D06 |
| GPLIN_000308900 | Parasitic | Member of effector gene family similar to *H. glycines* effector 4D06 |
| GPLIN_000309000 | Parasitic | Member of effector gene family similar to *H. glycines* effector 4D06 |
| GPLIN_000481100 | Parasitic | Member of effector gene family similar to *H. glycines* effector 4D06 |
| GPLIN_000792900 | Parasitic | Member of effector gene family similar to *H. glycines* effector 4D06 |
| GPLIN_000796500 | Parasitic | Member of effector gene family similar to *H. glycines* effector 4D06 |
| GPLIN_000803500 | Parasitic | Member of effector gene family similar to *H. glycines* effector 4D06 |
| GPLIN_000860700 | Parasitic | Member of effector gene family similar to *H. glycines* effector 4D06 |
| GPLIN_000912100 | Parasitic | Member of effector gene family similar to *H. glycines* effector 4D06 |
| GPLIN_000950100 | Parasitic | Member of effector gene family similar to *H. glycines* effector 4D06 |
| GPLIN_000950600 | Parasitic | Member of effector gene family similar to *H. glycines* effector 4D06 |
| GPLIN_000969800 | Parasitic | Member of effector gene family similar to *H. glycines* effector 4D06 |
| GPLIN_000969900 | Parasitic | Member of effector gene family similar to *H. glycines* effector 4D06 |
| GPLIN_000970000 | Parasitic | Member of effector gene family similar to *H. glycines* effector 4D06 |
| GPLIN_000970100 | Parasitic | Member of effector gene family similar to *H. glycines* effector 4D06 |
| GPLIN_001030900 | Parasitic | Member of effector gene family similar to *H. glycines* effector 4D06 |
| GPLIN_001038900 | Parasitic | Similar to *H. glycines* G18H08 effector |
| GPLIN_001038900 | Parasitic | Member of effector gene family similar to *H. glycines* effector 4D06 |
| GPLIN_001162100 | Parasitic | Member of effector gene family similar to *H. glycines* effector 4D06 |
| GPLIN_001221800 | Parasitic | Member of effector gene family similar to *H. glycines* effector 4D06 |
| GPLIN_001221900 | Parasitic | Member of effector gene family similar to *H. glycines* effector 4D06 |
| GPLIN_001255700 | Parasitic | Member of effector gene family similar to *H. glycines* effector 4D06 |
| GPLIN_001390400 | Parasitic | Member of effector gene family similar to *H. glycines* effector 4D06 |
| GPLIN_001390500 | Parasitic | Member of effector gene family similar to *H. glycines* effector 4D06 |
| GPLIN_001471200 | Parasitic | Member of effector gene family similar to *H. glycines* effector 4D06 |
| GPLIN_001582700 | Parasitic | Member of effector gene family similar to *H. glycines* effector 4D06 |
| GPLIN_001596100 | Parasitic | Member of effector gene family similar to *H. glycines* effector 4D06 |
| GPLIN_001606400 | Parasitic | Member of effector gene family similar to *H. glycines* effector 4D06 |
| GPLIN_001043600 | Parasitic | Member of 747 effector gene family |
| GPLIN_001090500 | Parasitic | Member of CLE effector protein family |
| GPLIN_000697600 | Parasitic | Member of CLE effector protein family, 4 CLE repeats |
| GPLIN_000167300 | Parasitic | Possible orthologue of *H. glycines* G10A06 effector; similarity to E3 Ligases |
| GPLIN_000188200 | Parasitic | Putative effector similar to *H. avenae*  gland cell protein |
| GPLIN_000349200 | Parasitic | Putative effector similar to *H. avenae*  gland cell protein and *H. glycines* effector Hgg 20 |
| GPLIN_000107400 | Parasitic | Putative effector similar to *H. glycines* Hgg17 effector |
| GPLIN_000120300 | Parasitic | Similar to *H. glycines* 30G12 effector |
| GPLIN_000637900 | Parasitic | Similar to *H. glycines* 30G12 effector |
| GPLIN_000638800 | Parasitic | Similar to *H. glycines* 30G12 effector |
| GPLIN_000668600 | Parasitic | Similar to *H. glycines* 30G12 effector |
| GPLIN_001339200 | Parasitic | Similar to *H. glycines* 30G12 effector |
| GPLIN_000370900 | Parasitic | Similar to *H. glycines* effector G19B10 |
| GPLIN_001416500 | Parasitic | Similar to *H. glycines* effector G19B10 |
| GPLIN_000763000 | Parasitic | Similar to *H. glycines* effector G23G11 |
| GPLIN_000667500 | Parasitic | Similar to *H. glycines* G4G05 and 30G12 effectors |
| GPLIN_000442900 | Constitutive | Contains *G. pallida*  orthologue of *H. glycines* G8A07 effector |
| GPLIN_000015300 | Constitutive | *G. pallida*  homologue of *H. glycines* effector G7E05 |
| GPLIN_001203000 | Constitutive | *G. pallida*  homologue of *H. glycines* effector 10C02 |
| GPLIN_000393900 | Constitutive | Large protein includes sequence similar to *H. glycines* effector scn1120 |
| GPLIN_000388900 | Constitutive | Member of effector gene family similar to *H. glycines* effector 4D06 |
| GPLIN_001358800 | Constitutive | Member of effector gene family similar to *H. glycines* effector 4D06 |
| GPLIN_001431400 | Constitutive | Member of effector gene family similar to *H. glycines* effector 4D06 |
| GPLIN_001443600 | Constitutive | Member of effector gene family similar to *H. glycines* effector 4D06 |
| GPLIN_000812600 | Constitutive | Member of 747 effector gene family |
| GPLIN_000950800 | Constitutive | Member of CLE effector protein family, one CLE motif |
| GPLIN_001090600 | Constitutive | Member of CLE effector protein family, one CLE motif |
| GPLIN_000785400 | Constitutive | Possible orthologue of *H. glycines* G10A06 effector; similarity to E3 Ligases |
| GPLIN_000760900 | Constitutive | Similar to *G. rostochiensis* E9 effector protein |
| GPLIN_000574800 | Constitutive | Similar to *H. glycines* effector gland cell secretory protein 3. Contains thioredoxin-like domain |
| GPLIN_000990400 | Constitutive | Similar to *H. glycines* effector gland cell secretory protein 3. Contains thioredoxin-like domain |
| GPLIN_001205000 | Constitutive | Similar to *H. glycines* effector gland cell secretory protein 3. Contains thioredoxin-like domain |
| GPLIN_000869800 | Constitutive | Similar to *H. glycines* secretory protein 11 putative effector. Similar to transthyretin-like proteins |
| GPLIN_000870000 | Constitutive | Similar to *H. glycines* secretory protein 11 putative effector. Similar to transthyretin-like proteins |
| GPLIN_000169700 | Constitutive | Similar to *H. glycines* secretory protein 12 putative effector. Similar to metalloprotease inhibitor |
| GPLIN_000621200 | Constitutive | Similar to *H. glycines* secretory protein 8 putative effector |
| GPLIN_000248100 | Parasitic and male | Similar to *H. glycines* effector G16A01 |
| GPLIN_000931100 | Parasitic and male | Member of 747 effector gene family |
| GPLIN_000933000 | Parasitic and male | Similar to *H. glycines* effector G17G01 |
| GPLIN_001384700 | Parasitic and male | Putative effector similar to *H. glycines* esophageal gland cell protein Hgg-20. Contains Kinase domain |

Supplementary Table 3: *G. pallida* genes similar to effectors characterised from other nematodes (excluding cell wall degrading and modifying enzymes). Expression profiles of each gene as inferred from RNAseq analysis are indicated.
