# Supplementary material for: Genomic characterisation of the effector complement of the potato cyst nematode Globodera pallida
Source: BMC Genomics. 2014 Oct 23;15(1):923. doi: 10.1186/1471-2164-15-923 (PMC4213498; doi:10.1186/1471-2164-15-923)
Supplement: Supplementary file 2 — Additional file 2: Table S4: Previously identified effectors from other cyst nematodes absent from the current G. pallida genome assembly. (DOCX 15 KB) [file 12864_2014_6605_MOESM2_ESM.docx]

| **Effectors not present in *G. pallida***  **predicted proteins** | **First identified in** | **Present in current**  **genome assembly or transcriptome** |
| --- | --- | --- |
| AF345801_1 | *H. glycines* | No |
| Hgg-25 | *H. glycines* | No |
| G16A01 | *H. glycines* | No |
| AF273728_1 gland cell secretory protein 1 | *H. glycines* | No |
| AF273733_1 gland cell secretory protein 6 | *H. glycines* | Transcriptome |
| Gland cell secretory protein 10 | *H. glycines* | No |
| Gland cell secretory protein 9 | *H. glycines* | No |
| Gland cell secretory protein 5 | *H. glycines* | No |
| Gland cell secretory protein 2 | *H. glycines* | No |
| Hgg-26 | *H. glycines* | No |
| G30C02 | *H. glycines* | No |
| G34B08 | *H. glycines* | No |
| G23G12 | *H. glycines* | No |
| G21E12 | *H. glycines* | No |
| G30D08 | *H. glycines* | No |
| G28B03 | *H. glycines* | Transcriptome |
| G8H07 | *H. glycines* | Transcriptome |
| G18H08 | *H. glycines* | No |
| G17G06 | *H. glycines* | No |
| AF345800_1 SCN secretory protein | *H. glycines* | No |
| *G. pallida* “66P1” gene family | *G. rostochiensis* | No – present in previous assemblies and in transcriptome |
| 747_22_2 | *G. rostochiensis* | Transcriptome |
| A42 | *G. rostochiensis* | Yes – uncalled. Present in transcriptome |

**Supplementary table 4.** Previously identified effectors from other cyst nematodes absent from the current *G. pallida* genome assembly.
