# Supplementary material for: Genomic characterisation of the effector complement of the potato cyst nematode Globodera pallida
Source: BMC Genomics. 2014 Oct 23;15(1):923. doi: 10.1186/1471-2164-15-923 (PMC4213498; doi:10.1186/1471-2164-15-923)
Supplement: Supplementary file 3 — Additional file 3: Table S5: Globodera pallida secreted proteins up-regulated in J2 or early parasitic stages that may represent novel effector candidates. (DOCX 16 KB) [file 12864_2014_6605_MOESM3_ESM.docx]

| GPLIN_000948600 | GPLIN_001463000 | GPLIN_000834600 |
| --- | --- | --- |
| GPLIN_001318000 | GPLIN_000847100 | GPLIN_000028200 |
| GPLIN_000319500 | GPLIN_000342300 | GPLIN_001232800 |
| GPLIN_001185000 | GPLIN_001263700 | GPLIN_000466900 |
| GPLIN_001268500 | GPLIN_000361100 | GPLIN_001391000 |
| GPLIN_000510600 | GPLIN_000744000 | GPLIN_000318900 |
| GPLIN_000957300 | GPLIN_000555400 | GPLIN_001008400 |
| GPLIN_001016900 | GPLIN_000208800 | GPLIN_001138500 |
| GPLIN_000927400 | GPLIN_000027900 | GPLIN_000142200 |
| GPLIN_000357600 | GPLIN_000886700 | GPLIN_000187400 |
| GPLIN_001262300 | GPLIN_000228700 | GPLIN_001335500 |
| GPLIN_000061100 | GPLIN_000063700 | GPLIN_000608100 |
| GPLIN_000713500 | GPLIN_001196900 | GPLIN_000897000 |
| GPLIN_000943100 | GPLIN_001153300 | GPLIN_000819000 |
| GPLIN_000172000 | GPLIN_000897600 | GPLIN_001127400 |
| GPLIN_000776900 | GPLIN_001004000 | GPLIN_000966000 |
| GPLIN_000126000 | GPLIN_001223000 | GPLIN_000886500 |
| GPLIN_000919700 | GPLIN_000609400 | GPLIN_000122100 |
| GPLIN_000723200 | GPLIN_000376600 | GPLIN_001080000 |
| GPLIN_000280900 | GPLIN_000281300 | GPLIN_000516100 |
| GPLIN_000495300 | GPLIN_000818900 | GPLIN_000271900 |
| GPLIN_000185800 | GPLIN_001244900 | GPLIN_000167000 |
| GPLIN_000424400 | GPLIN_000100500 | GPLIN_001030400 |
| GPLIN_001344300 | GPLIN_000886600 | GPLIN_000698800 |
| GPLIN_000283500 | GPLIN_000208700 | GPLIN_000195900 |
| GPLIN_001066900 | GPLIN_001099200 | GPLIN_001030700 |
| GPLIN_000120500 | GPLIN_000614900 | GPLIN_000589200 |
| GPLIN_001040900 | GPLIN_000641200 | GPLIN_001138300 |
| GPLIN_001031700 | GPLIN_000696300 | GPLIN_000689500 |
| GPLIN_001417900 | GPLIN_001184500 | GPLIN_000610000 |
| GPLIN_001319300 | GPLIN_000758500 | GPLIN_001304400 |
| GPLIN_000943000 | GPLIN_000187600 | GPLIN_001183800 |
| GPLIN_000333100 | GPLIN_000063100 | GPLIN_000241600 |
| GPLIN_000616800 | GPLIN_000319000 | GPLIN_001550200 |
| GPLIN_000333000 | GPLIN_000807000 | GPLIN_000140200 |
| GPLIN_001153200 | GPLIN_001138700 | GPLIN_000821100 |
| GPLIN_001592300 | GPLIN_000560800 | GPLIN_000258900 |
| GPLIN_001292400 | GPLIN_000758200 | GPLIN_001146800 |
| GPLIN_000075700 | GPLIN_000209100 | GPLIN_000925000 |

**Supplementary Table 5:** *Globodera pallida* secreted proteins up-regulated in J2 or early parasitic stages that may represent novel effector candidates.
