# Supplementary figures and images for: Genomic characterisation of the effector complement of the potato cyst nematode Globodera pallida
Source: BMC Genomics. 2014 Oct 23;15(1):923. doi: 10.1186/1471-2164-15-923 (PMC4213498; doi:10.1186/1471-2164-15-923)

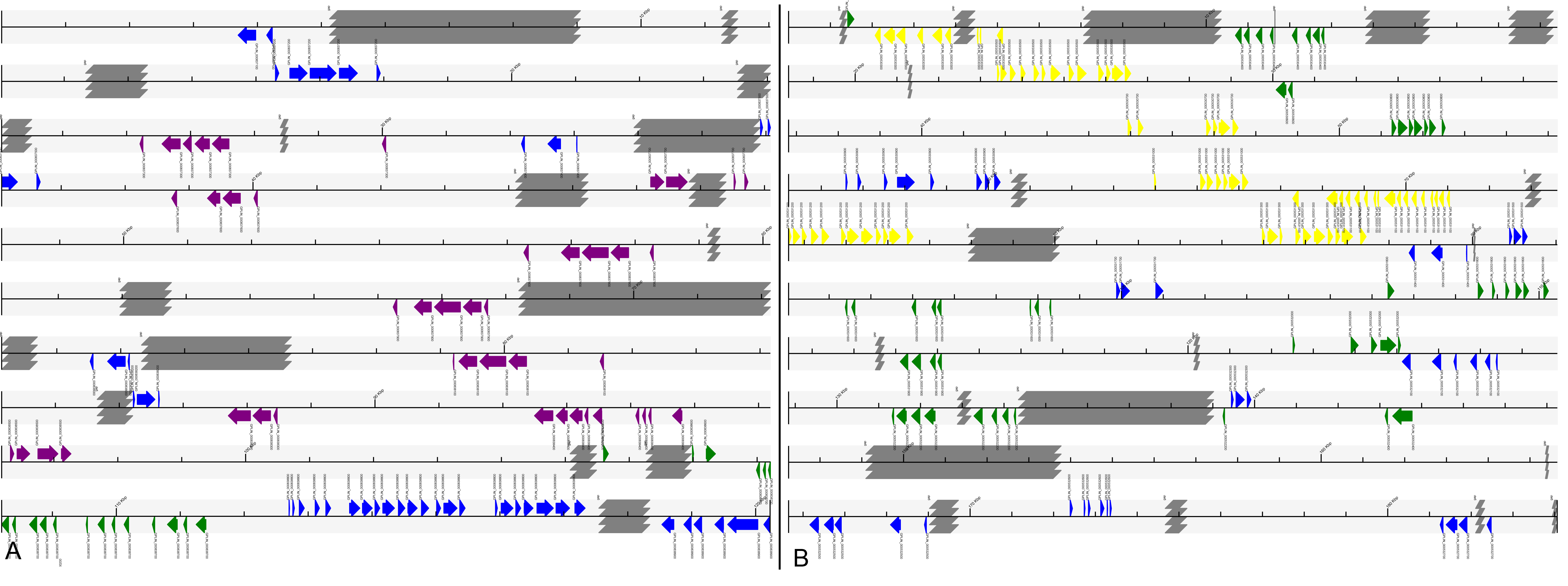

Supplement: Supplementary file 4 — Additional file 4: Figure S1: Locations of exons (coloured arrows) in scaffolds 299 (A) and 141 (B). Exons of genes encoding proteins similar to “H. avenae dorsal gland cell protein” are indicated in purple on panel A and exons of genes encoding proteins that contain a SPRY domain are indicated in yellow on panel B. Other predicted genes are indicated in blue or green (alternating genes). Direction of arrows indicates orientation of predicted open reading frames. Grey shading indicates unsequenced regions of scaffolds. (PNG 495 KB) [file 12864_2014_6605_MOESM4_ESM.png]

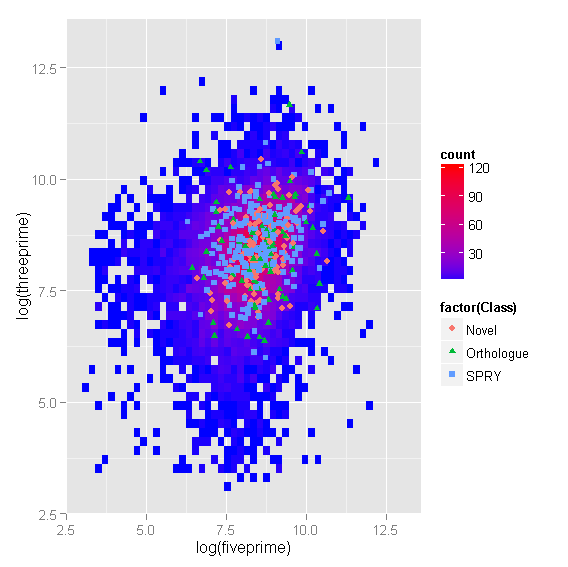

Supplement: Supplementary file 5 — Additional file 5: Figure S2: Distribution of various effector groups across gene sparse and gene rich regions of the Globodera pallida genome. Heat map generated reflecting gene density and the distribution of three classes of effector candidates: the SPRYSECs, G. pallida orthologs of effectors from other PPN and “novel” effectors. (PNG 12 KB) [file 12864_2014_6605_MOESM5_ESM.png]

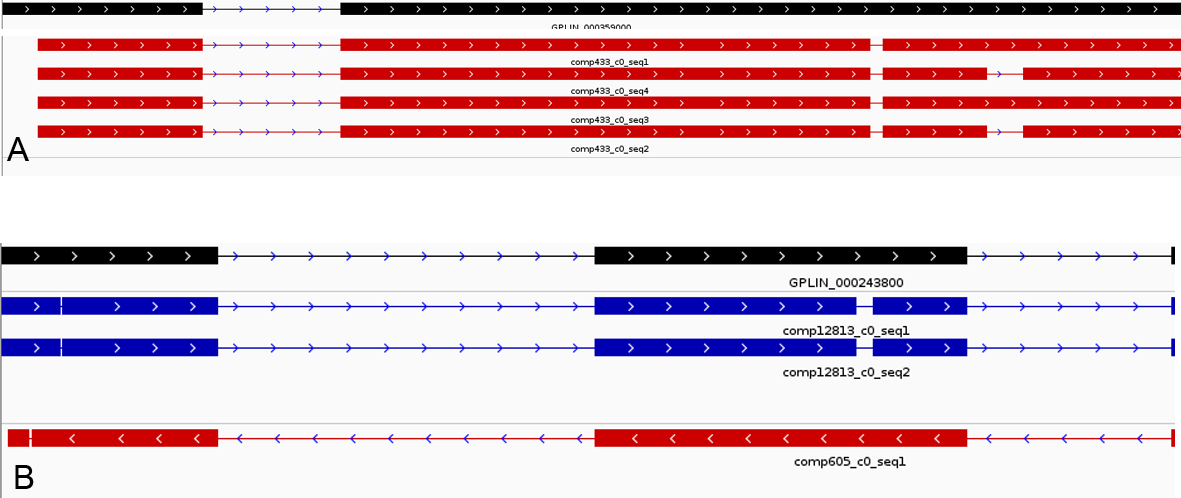

Supplement: Supplementary file 6 — Additional file 6: Figure S3: Example of potential alternative splicing events in G. pallida effectors Figure S3A: Alternative splicing of GPLIN_000359000 within one life stage. Black bar indicates predicted sequence from genome with bars showing predicted coding region from gene model; introns are shown as lines. Red bars indicate de novo assembled transcripts from RNA extracted from parasitic nematodes 7dpi, red lines indicate gaps compared to genome sequence. Figure S3B: Alternative splicing of GPLIN_000243800 between life stages. Black bar indicates predicted sequence from genome with bars showing predicted coding region from gene model; introns are shown as lines. Red and blue bars/lines indicate de novo assembled transcripts from parasitic nematodes 7dpi and J2s respectively. (TIFF 2 MB) [file 12864_2014_6605_MOESM6_ESM.tiff]
