# Supplementary material for: Genomic characterisation of the effector complement of the potato cyst nematode Globodera pallida
Source: BMC Genomics. 2014 Oct 23;15(1):923. doi: 10.1186/1471-2164-15-923 (PMC4213498; doi:10.1186/1471-2164-15-923)
Supplement: Supplementary file 7 — Additional file 7: Table S1: Primers used to generate DNA fragments used for synthesis of probes for in situ hybridisation. (DOCX 14 KB) [file 12864_2014_6605_MOESM7_ESM.docx]

| Name | Sequence |
| --- | --- |
| GPLIN_001203000F | CAAGGCCGAAGCTGAAGCC |
| GPLIN_001203000R | ATGCTTAGGCTTCTTTCCGC |
| GPLIN_000854400F | TATCCGAGTCCTTCACTACTG |
| GPLIN_000854400R | AAGATGATCATCCAGTCCAAG |
| GPLIN_000662500F | ACCGCAATACAGACGATGATGG |
| GPLIN_000662500R | GCTGTCGGGAGTTTGTCACAC |
| GPLIN_000235400F | ACCATCCAGTGCTCCAGTTGT |
| GPLIN_000235400R | TTCGTCCATATTGGATTTTGG |
| GPLIN_000457000F | AAGGAGCACAAAGAGCCTGC |
| GPLIN_000457000R | AAATGTTCTCGAAGATGGACG |
| A42_ISH_F | TGTGGTGGTGACTGTTTTGG |
| A42_ISH_R | TCGTCTTATGAGCTTGCTTC |
| GPLIN_000201400F | CACGATTTCAACATACTTACT |
| GPLIN_000201400R | TTCCTGCTCGTTCGGCTTGAG |
| GPLIN_001082900F | TTTTACACGCTGAACGGAGA |
| GPLIN_001082900R | ACAATGTTTGAATTCAAAATTCGG |
| GPLIN_000657200F | TCGCCAAAACCGTCAACATC |
| GPLIN_000657200R | AGCTCAGAAGGTGTGGAGTC |
| GPLIN_000834600F | CTGCCCAACCTTCTCGTAAC |
| GPLIN_000834600R | GTGCATTTGCCTTTTCCAGT |
| GPLIN_000333000F | ACTTTGCGAGTGGCACTTTT |
| GPLIN_000333000R | CTGGTGGTTTGCTTTCATGG |

**Supplementary Table 1:** Primers used to generate DNA fragments used for synthesis of probes for *in situ* hybridisation.
