# Supplementary material for: Genomic characterisation of the effector complement of the potato cyst nematode Globodera pallida
Source: BMC Genomics. 2014 Oct 23;15(1):923. doi: 10.1186/1471-2164-15-923 (PMC4213498; doi:10.1186/1471-2164-15-923)
Supplement: Supplementary file 8 — Additional file 8: Table S2: Sequences of primers used for cloning full length effectors (without signal peptides). (DOCX 14 KB) [file 12864_2014_6605_MOESM8_ESM.docx]

| Primer name | Sequence | Purpose |
| --- | --- | --- |
| eGFPF | ACCATGGTGAGCAAGGGC | Cloning eGFP |
| eGFPR | TCACTTGTACAGCTCGTCCATG |  |
| GPLIN_000015300F | ACCATGTTCCATTGCTGGGATTCGA | Cloning 00015300 |
| GPLIN_000015300R | TCAATTTGGTCCGTTGCACAGC |  |
| GpA42F | ACCATGTGTGGTGGTGACTGTTTTGG | Cloning Gp A42 |
| GpA42R | TCATTTTCGTCTTATGAGCTTGCTTC |  |
| GPLIN_000235400F | ACCATGGCTCTTCTGGACACGGGTC | Cloning 235400 |
| GPLIN_000235400R | TCATTCGTCCATATTGGATTTTGG |  |
| GPLIN_000662500F | ACCATGACACCTAACGATAACCCGATTG | Cloning 662500 |
| GPLIN_000662500R | TCAAGCACAGAAAGGCGAAAAGA |  |
| GPLIN_000457000F | ACCATGCAAGATGATGATGACAAAGATGC | Cloning 457000 |
| GPLIN_000457000R | TCAGTTCTTGCCAAGCCCAATT |  |
| GPLIN_001465500F | ACCATGAATGAGTTCAAAATGAACGAGCAAC | Cloning 1465500 |
| GPLIN_001465500R | CTAAATGCCATCGGCAAAGTTGTATTTG |  |

**Supplementary Table 2:** Sequences of primers used for cloning full length effectors (without signal peptides).
